# Redetermination of 3,5-dimethyl­phenol

**DOI:** 10.1107/S1600536811013547

**Published:** 2011-04-16

**Authors:** Richard Betz, Cedric McCleland, Harold Marchand

**Affiliations:** a Nelson Mandela Metropolitan University, Summerstrand Campus, Department of Chemistry, University Way, Summerstrand, PO Box 77000, Port Elizabeth 6031, South Africa

## Abstract

The previous structure determination [Gillier-Pandraud *et al.* (1972 ▶). *C. R. Acad. Sci. Ser. C*, **275**, 1495] of the title compound, C_8_H_10_O, did not report atomic coordinates. There are two mol­ecules in the asymmetric unit, *A* and *B*, which both show approximate non-crystallographic *C*_s_ symmetry. The intra­cyclic C—C—C angles cover the range 118.74 (12)–121.76 (13)°. In the crystal, mol­ecules are linked by O—H⋯O hydrogen bonds, generating [001] *C*_2_^2^(4) chains such that mol­ecules *A* and *B* alternate. There is no aromatic π–π stacking in the crystal as the shortest centroid–centroid distance is greater than 4.74 Å.

## Related literature

The compound has been deposited with the CSD (refcode: DMPHNL) but no three-dimensional-coordinates are available (Gillier-Pandraud *et al.*, 1972 ▶). For graph-set analysis of hydrogen bonds, see: Etter *et al.* (1990 ▶); Bernstein *et al.* (1995 ▶).
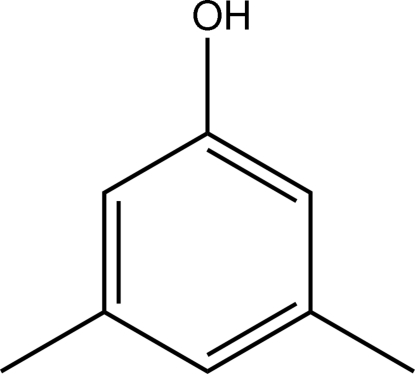


## Experimental

### 

#### Crystal data

C_8_H_10_O
*M* *_r_* = 122.16
Monoclinic, 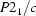

*a* = 11.9807 (6) Å
*b* = 13.8725 (7) Å
*c* = 8.5378 (4) Å
β = 90.000 (2)°
*V* = 1419.00 (12) Å^3^
*Z* = 8
Mo *K*α radiation
μ = 0.07 mm^−1^
*T* = 200 K
0.50 × 0.41 × 0.33 mm

#### Data collection

Bruker APEXII CCD diffractometer
12884 measured reflections
3392 independent reflections
2998 reflections with *I* > 2σ(*I*)
*R*_int_ = 0.031

#### Refinement

*R*[*F* ^2^ > 2σ(*F* ^2^)] = 0.051
*wR*(*F* ^2^) = 0.143
*S* = 1.04
3392 reflections
169 parameters
H-atom parameters constrained
Δρ_max_ = 0.30 e Å^−3^
Δρ_min_ = −0.25 e Å^−3^

### 

Data collection: *APEX2* (Bruker, 2010 ▶); cell refinement: *SAINT* (Bruker, 2010 ▶); data reduction: *SAINT*; program(s) used to solve structure: *SHELXS97* (Sheldrick, 2008 ▶); program(s) used to refine structure: *SHELXL97* (Sheldrick, 2008 ▶); molecular graphics: *ORTEP-3* (Farrugia, 1997 ▶) and *Mercury* (Macrae *et al.*, 2006 ▶); software used to prepare material for publication: *SHELXL97* and *PLATON* (Spek, 2009 ▶).

## Supplementary Material

Crystal structure: contains datablocks I, global. DOI: 10.1107/S1600536811013547/hb5838sup1.cif

Structure factors: contains datablocks I. DOI: 10.1107/S1600536811013547/hb5838Isup2.hkl

Additional supplementary materials:  crystallographic information; 3D view; checkCIF report

## Figures and Tables

**Table 1:** Hydrogen-bond geometry (Å, °)

*D*—H⋯*A*	*D*—H	H⋯*A*	*D*⋯*A*	*D*—H⋯*A*
O1—H1⋯O2^i^	0.84	1.91	2.7463 (13)	171
O2—H2⋯O1^ii^	0.84	1.90	2.7327 (13)	172

Symmetry codes: (i) 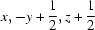; (ii) 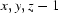.

**Fig. 1.**
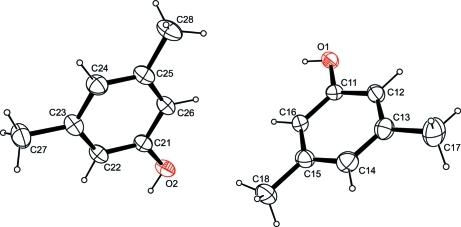
The molecular structure of the title compound, with anisotropic displacement ellipsoids drawn at 50% probability level.

**Fig. 2.**
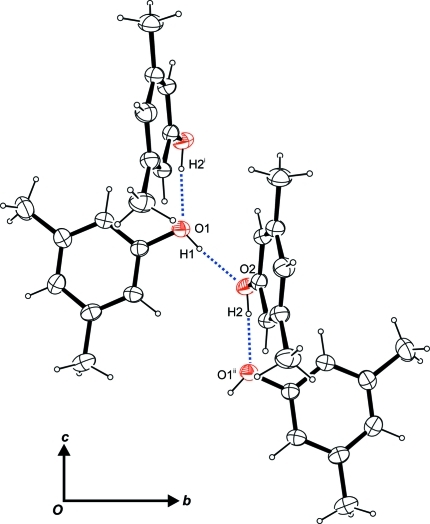
Intermolecular contacts, viewed along [-1 0 0]. Symmetry operators: ix, y, z + 1; iix, y, z - 1.

**Fig. 3.**
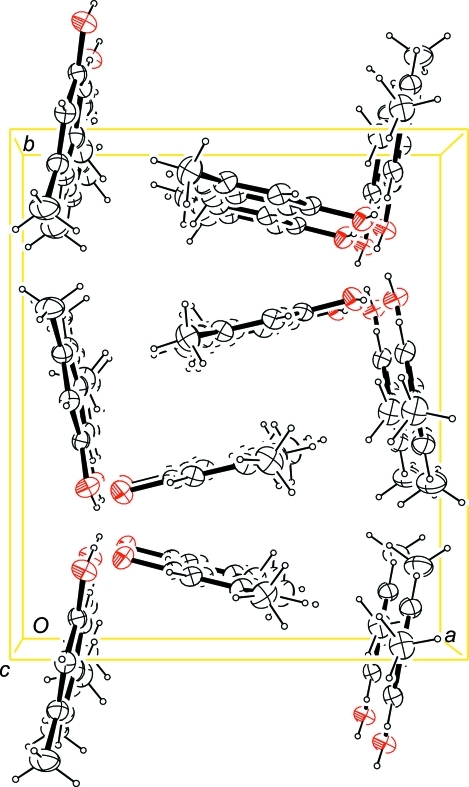
Molecular packing of the title compound, viewed along [0 0 - 1] (anisotropic displacement ellipsoids drawn at 50% probability level).

## supplementary crystallographic information

### Comment

Phenol and derivatives are interesting bonding partners for a variety of transition metals and elements from the *p*-block of the periodic system. They can act as neutral or – upon deprotonation – as anionic monodentate ligands. Upon variation of the substituents on the aromatic system, a seemingly endless series of symmetric as well as asymmetric phenol derivatives featuring different steric pretenses and acidities of the hydroxyl-group are available. At the beginning of a larger study aimed at elucidating the coordination behaviour of various phenol-derivatives in dependence of pH-value and substitution pattern on the phenyl moiety, it seemed of interest to determine the crystal structure of the title compound to enable comparisons with metric parameters in envisioned coordination compounds. Although the structure has been deposited with the Cambridge Structural Database (Gillier-Pandraud *et al.*, 1972), no three-dimensional-coordinates were provided.

The asymmetric unit comprises two molecules of the title compound which are nearly orientated perpendicular to each other. The least-squares planes defined by the C-atoms of the respective phenyl moieties intersect at an angle of 87.87 (4) °. Intracyclic C–C–C angles span a range of 119–122 ° with the biggest angles invariably found on the C-atoms bearing the hydroxyl group and the C-atoms in *para*-position to these, respectively. The H-atoms of both hydroxyl groups are approximately in plane with the aromatic systems (Fig. 1).

In the crystal structure, a set of cooperative hydrogen bonds connects the molecules to infinite chains along the crystallographic *c*-axis. Both molecules in the asymmetric unit participate alternately in these chains. In terms of graph-set analysis, the description of these intermolecular interactions necessitates a *C*^2^_2_(4) descriptor on the binary level (Fig. 2). The closest distance between two centers of gravity was measured at 4.7437 (8) Å.

The packing of the title compound in the crystal structure is shown in Figure 3.

### Experimental

The compound was obtained commercially (Fluka). Crystals suitable for the X-ray diffraction study were taken directly from the provided compound.

### Refinement

Carbon-bound H-atoms were placed in calculated positions (C—H 0.98 Å for the methyl groups and C—H 0.95 Å for aromatic carbon atoms) and were included in the refinement in the riding model approximation, with *U*(H) set to 1.5*U*_eq_(C) for the methyl groups and 1.2*U*_eq_(C) for aromatic carbon atoms. The H atoms of the methyl groups were allowed to rotate with a fixed angle around the C—C bonds to best fit the experimental electron density (HFIX 137 in the *SHELX* program suite (Sheldrick, 2008)). The H atom of the hydroxyl groups were allowed to rotate with a fixed angle around the O—C bonds to best fit the experimental electron density (HFIX 147 in the *SHELX* program suite (Sheldrick, 2008)), their *U*(H) set to 1.5*U*_eq_(O).

### Crystal data

**Table:**

C_8_H_10_O	*F*(000) = 528
*M**_r_* = 122.16	*D*_x_ = 1.144 Mg m^−^^3^
Monoclinic, *P*2_1_/*c*	Mo *K*α radiation, λ = 0.71073 Å
Hall symbol: -P 2ybc	Cell parameters from 8727 reflections
*a* = 11.9807 (6) Å	θ = 2.3–28.3°
*b* = 13.8725 (7) Å	µ = 0.07 mm^−^^1^
*c* = 8.5378 (4) Å	*T* = 200 K
β = 90.000 (2)°	Block, colourless
*V* = 1419.00 (12) Å^3^	0.50 × 0.41 × 0.33 mm
*Z* = 8	

### Data collection

**Table:**

Bruker APEXII CCD diffractometer	2998 reflections with *I* > 2σ(*I*)
Radiation source: fine-focus sealed tube	*R*_int_ = 0.031
graphite	θ_max_ = 28.0°, θ_min_ = 3.3°
φ and ω scans	*h* = −15→9
12884 measured reflections	*k* = −18→18
3392 independent reflections	*l* = −11→11

### Refinement

**Table:**

Refinement on *F*^2^	Primary atom site location: structure-invariant direct methods
Least-squares matrix: full	Secondary atom site location: difference Fourier map
*R*[*F*^2^ > 2σ(*F*^2^)] = 0.051	Hydrogen site location: inferred from neighbouring sites
*wR*(*F*^2^) = 0.143	H-atom parameters constrained
*S* = 1.04	*w* = 1/[σ^2^(*F*_o_^2^) + (0.0724*P*)^2^ + 0.556*P*] where *P* = (*F*_o_^2^ + 2*F*_c_^2^)/3
3392 reflections	(Δ/σ)_max_ < 0.001
169 parameters	Δρ_max_ = 0.30 e Å^−^^3^
0 restraints	Δρ_min_ = −0.25 e Å^−^^3^

### Fractional atomic coordinates and isotropic or equivalent isotropic displacement parameters (Å^2^)

**Table:**

	*x*	*y*	*z*	*U*_iso_*/*U*_eq_	
O1	0.16676 (9)	0.32297 (7)	0.83341 (11)	0.0322 (2)	
H1	0.1864	0.2833	0.7644	0.048*	
C11	0.15120 (10)	0.41221 (9)	0.76595 (14)	0.0263 (3)	
C12	0.12708 (11)	0.48876 (10)	0.86483 (15)	0.0294 (3)	
H12	0.1224	0.4787	0.9747	0.035*	
C13	0.10982 (12)	0.58017 (10)	0.80279 (17)	0.0334 (3)	
C14	0.11818 (12)	0.59291 (10)	0.64172 (17)	0.0356 (3)	
H14	0.1070	0.6554	0.5989	0.043*	
C15	0.14249 (11)	0.51675 (10)	0.54173 (15)	0.0320 (3)	
C16	0.15847 (11)	0.42544 (10)	0.60545 (14)	0.0292 (3)	
H16	0.1744	0.3723	0.5390	0.035*	
C17	0.08510 (17)	0.66459 (12)	0.9083 (2)	0.0502 (4)	
H171	0.0653	0.6410	1.0129	0.075*	
H172	0.0227	0.7017	0.8651	0.075*	
H173	0.1512	0.7059	0.9155	0.075*	
C18	0.15133 (14)	0.53221 (12)	0.36751 (17)	0.0419 (4)	
H181	0.0772	0.5460	0.3246	0.063*	
H182	0.1813	0.4739	0.3181	0.063*	
H183	0.2012	0.5867	0.3465	0.063*	
O2	0.24467 (8)	0.31707 (7)	0.13407 (11)	0.0327 (2)	
H2	0.2264	0.3213	0.0393	0.049*	
C21	0.35593 (11)	0.34152 (8)	0.15086 (14)	0.0269 (3)	
C22	0.42454 (12)	0.35771 (9)	0.02291 (14)	0.0293 (3)	
H22	0.3951	0.3537	−0.0802	0.035*	
C23	0.53654 (12)	0.37975 (10)	0.04558 (15)	0.0321 (3)	
C24	0.57689 (12)	0.38687 (10)	0.19756 (16)	0.0341 (3)	
H24	0.6532	0.4026	0.2140	0.041*	
C25	0.50780 (12)	0.37150 (9)	0.32656 (15)	0.0318 (3)	
C26	0.39652 (12)	0.34879 (9)	0.30206 (14)	0.0295 (3)	
H26	0.3483	0.3383	0.3887	0.035*	
C27	0.61218 (14)	0.39382 (12)	−0.09337 (17)	0.0418 (4)	
H271	0.6191	0.3330	−0.1508	0.063*	
H272	0.6861	0.4145	−0.0572	0.063*	
H273	0.5807	0.4432	−0.1625	0.063*	
C28	0.55365 (15)	0.37856 (12)	0.49024 (17)	0.0434 (4)	
H281	0.4918	0.3812	0.5652	0.065*	
H282	0.5989	0.4371	0.4998	0.065*	
H283	0.6001	0.3220	0.5123	0.065*	

### Atomic displacement parameters (Å^2^)

**Table:**

	*U*^11^	*U*^22^	*U*^33^	*U*^12^	*U*^13^	*U*^23^
O1	0.0423 (6)	0.0306 (5)	0.0236 (4)	0.0077 (4)	−0.0022 (4)	−0.0006 (3)
C11	0.0237 (6)	0.0303 (6)	0.0249 (6)	0.0020 (5)	−0.0022 (4)	0.0009 (5)
C12	0.0298 (6)	0.0339 (6)	0.0244 (6)	−0.0006 (5)	−0.0002 (5)	−0.0023 (5)
C13	0.0327 (7)	0.0312 (6)	0.0362 (7)	−0.0009 (5)	0.0021 (5)	−0.0036 (5)
C14	0.0360 (7)	0.0316 (6)	0.0392 (7)	0.0001 (5)	0.0024 (6)	0.0060 (5)
C15	0.0268 (6)	0.0412 (7)	0.0281 (6)	−0.0004 (5)	0.0006 (5)	0.0054 (5)
C16	0.0279 (6)	0.0360 (6)	0.0238 (6)	0.0041 (5)	0.0006 (5)	−0.0016 (5)
C17	0.0630 (11)	0.0348 (8)	0.0526 (10)	0.0025 (7)	0.0076 (8)	−0.0098 (7)
C18	0.0440 (8)	0.0520 (9)	0.0297 (7)	0.0017 (7)	0.0027 (6)	0.0105 (6)
O2	0.0341 (5)	0.0389 (5)	0.0252 (4)	−0.0077 (4)	−0.0050 (4)	0.0058 (4)
C21	0.0318 (7)	0.0234 (5)	0.0255 (6)	−0.0033 (5)	−0.0018 (5)	0.0020 (4)
C22	0.0380 (7)	0.0294 (6)	0.0206 (5)	−0.0045 (5)	−0.0022 (5)	0.0000 (4)
C23	0.0381 (7)	0.0319 (6)	0.0261 (6)	−0.0051 (5)	0.0019 (5)	0.0000 (5)
C24	0.0326 (7)	0.0381 (7)	0.0315 (7)	−0.0070 (6)	−0.0038 (5)	0.0001 (5)
C25	0.0403 (8)	0.0305 (6)	0.0246 (6)	−0.0039 (5)	−0.0050 (5)	0.0002 (5)
C26	0.0377 (7)	0.0288 (6)	0.0220 (6)	−0.0032 (5)	0.0003 (5)	0.0020 (4)
C27	0.0408 (8)	0.0537 (9)	0.0309 (7)	−0.0084 (7)	0.0069 (6)	0.0002 (6)
C28	0.0518 (9)	0.0508 (9)	0.0278 (7)	−0.0088 (7)	−0.0122 (6)	−0.0003 (6)

### Geometric parameters (Å, °)

**Table:**

O1—C11	1.3780 (15)	O2—C21	1.3828 (16)
O1—H1	0.8400	O2—H2	0.8400
C11—C16	1.3853 (17)	C21—C26	1.3832 (17)
C11—C12	1.3871 (17)	C21—C22	1.3855 (18)
C12—C13	1.3897 (19)	C22—C23	1.390 (2)
C12—H12	0.9500	C22—H22	0.9500
C13—C14	1.390 (2)	C23—C24	1.3881 (19)
C13—C17	1.507 (2)	C23—C27	1.5056 (19)
C14—C15	1.389 (2)	C24—C25	1.3942 (19)
C14—H14	0.9500	C24—H24	0.9500
C15—C16	1.3918 (19)	C25—C26	1.386 (2)
C15—C18	1.5066 (18)	C25—C28	1.5048 (18)
C16—H16	0.9500	C26—H26	0.9500
C17—H171	0.9800	C27—H271	0.9800
C17—H172	0.9800	C27—H272	0.9800
C17—H173	0.9800	C27—H273	0.9800
C18—H181	0.9800	C28—H281	0.9800
C18—H182	0.9800	C28—H282	0.9800
C18—H183	0.9800	C28—H283	0.9800
			
C11—O1—H1	109.5	C21—O2—H2	109.5
O1—C11—C16	121.60 (11)	O2—C21—C26	116.97 (11)
O1—C11—C12	117.49 (11)	O2—C21—C22	122.00 (11)
C16—C11—C12	120.90 (12)	C26—C21—C22	121.03 (12)
C11—C12—C13	119.84 (12)	C21—C22—C23	119.91 (12)
C11—C12—H12	120.1	C21—C22—H22	120.0
C13—C12—H12	120.1	C23—C22—H22	120.0
C12—C13—C14	118.84 (12)	C24—C23—C22	118.82 (12)
C12—C13—C17	120.70 (13)	C24—C23—C27	121.18 (13)
C14—C13—C17	120.45 (13)	C22—C23—C27	119.99 (12)
C15—C14—C13	121.76 (13)	C23—C24—C25	121.38 (13)
C15—C14—H14	119.1	C23—C24—H24	119.3
C13—C14—H14	119.1	C25—C24—H24	119.3
C14—C15—C16	118.74 (12)	C26—C25—C24	119.13 (12)
C14—C15—C18	120.88 (13)	C26—C25—C28	120.41 (13)
C16—C15—C18	120.38 (13)	C24—C25—C28	120.46 (13)
C11—C16—C15	119.91 (12)	C21—C26—C25	119.71 (12)
C11—C16—H16	120.0	C21—C26—H26	120.1
C15—C16—H16	120.0	C25—C26—H26	120.1
C13—C17—H171	109.5	C23—C27—H271	109.5
C13—C17—H172	109.5	C23—C27—H272	109.5
H171—C17—H172	109.5	H271—C27—H272	109.5
C13—C17—H173	109.5	C23—C27—H273	109.5
H171—C17—H173	109.5	H271—C27—H273	109.5
H172—C17—H173	109.5	H272—C27—H273	109.5
C15—C18—H181	109.5	C25—C28—H281	109.5
C15—C18—H182	109.5	C25—C28—H282	109.5
H181—C18—H182	109.5	H281—C28—H282	109.5
C15—C18—H183	109.5	C25—C28—H283	109.5
H181—C18—H183	109.5	H281—C28—H283	109.5
H182—C18—H183	109.5	H282—C28—H283	109.5
			
O1—C11—C12—C13	−179.53 (12)	O2—C21—C22—C23	−178.39 (12)
C16—C11—C12—C13	0.0 (2)	C26—C21—C22—C23	1.3 (2)
C11—C12—C13—C14	−0.6 (2)	C21—C22—C23—C24	−1.2 (2)
C11—C12—C13—C17	−179.15 (14)	C21—C22—C23—C27	177.78 (13)
C12—C13—C14—C15	0.4 (2)	C22—C23—C24—C25	0.6 (2)
C17—C13—C14—C15	179.05 (14)	C27—C23—C24—C25	−178.36 (14)
C13—C14—C15—C16	0.2 (2)	C23—C24—C25—C26	−0.1 (2)
C13—C14—C15—C18	−179.91 (14)	C23—C24—C25—C28	179.34 (14)
O1—C11—C16—C15	−179.85 (12)	O2—C21—C26—C25	178.94 (12)
C12—C11—C16—C15	0.6 (2)	C22—C21—C26—C25	−0.76 (19)
C14—C15—C16—C11	−0.7 (2)	C24—C25—C26—C21	0.2 (2)
C18—C15—C16—C11	179.39 (13)	C28—C25—C26—C21	−179.28 (13)

### Hydrogen-bond geometry (Å, °)

**Table:**

*D*—H···*A*	*D*—H	H···*A*	*D*···*A*	*D*—H···*A*
O1—H1···O2^i^	0.84	1.91	2.7463 (13)	171
O2—H2···O1^ii^	0.84	1.90	2.7327 (13)	172

Symmetry codes: (i) *x*, −*y*+1/2, *z*+1/2; (ii) *x*, *y*, *z*−1.
